# The novel dithiocarbamate, DpdtC suppresses HER2-overexpressed cancer cells by up-regulating NDRG1 via inactivation of HER2-ERK 1/2 signaling

**DOI:** 10.1038/s41598-018-21768-1

**Published:** 2018-02-21

**Authors:** Yun Yang, Youxun Liu, Rui Guo, Yun Fu, Ziheng Zhang, Pengfei Zhang, Pingxin Zhou, Tingting Wang, Tengfei Huang, Xiaotong Li, Changzheng Li

**Affiliations:** 1 School of Basic Medical Sciences, Xinxiang Medical University, Xinxiang, China; 2 College of Biomedical Engineering, Xinxiang Medical University, Xinxiang, China; 3 School of Life Sciences, Xiamen University, Xiamen, China

## Abstract

Dithiocarbamate has been tested for its effective anti-tumor activity, but the underlying mechanism remains unclear. We previously prepared a novel diththiocarbamate derivative, DpdtC with an ability of catalase inhibition. Here, we for the first time investigated the growth inhibition effects of DpdtC on HER2-amplified cancer cells and elucidated its mechanism of action. Results showed that DpdtC exerted the potent anti-tumor effects against HER2-overexpressed SK-OV-3 and SK-BR-3 cells, especially on SK-OV-3 cells with a higher NDRG1 level, which was also confirmed in the SK-OV-3 xenograft model. Interestingly, we observed that NDRG1 was up-regulated, while membrane expression of HER2 was regressed in SK-OV-3 cells upon DpdtC treatment. In agreement, silencing endogenous NDRG1 also increased the expression of HER2 in SK-OV-3 cells, while overexpressing NDRG1 decreased HER2 expression in SK-BR-3 cells. Furthermore, our results showed the formation of the EGFR/HER2 heterodimer was attenuated and phosphorylation of ERK1/2 was inhibited in SK-OV-3 cells when treated with DpdtC. Collectively, these observations demonstrated that NDRG1 plays an important role in mediating the inhibition effects of DpdtC in HER2-overexpressed cancer cells via selective targeting of the HER2-ERK1/2 pathway. Hence, our investigation suggests that up-regulation of NDRG1 by DpdtC is a promising therapeutic approach in HER2-overexpressed cancers.

## Introduction

Metal chelators are promising therapeutic agents that show marked and selective anti-tumor activity^1,2^. As we know, cancer cells have an increased demand for iron and copper to maintain proper cell growth rate; therefore, the use of chelators for cancer treatment has been an potential option^3,4^. The iron chelators such as di-2-pyridyl ketone-4,4-dimethyl-3-thiosemicarbazone (Dp44mT) and desferrioxamine (DFO) have shown pronounced inhibitory effects in several types of cancer^5,6^. Dithiocarbamates constitute a group of sulfur-containing compounds with an effective chelating potency toward metal ions^7^, which can modulate the key molecules involved in important processes, such as apoptosis, oxidative stress, transcription, and degradation of proteins^3,8^. However, their molecular targets and mechanisms of action remain to be completely addressed.

NDRG1 belongs to the NDRG (N-myc downstream-regulated gene) family which has been reported to function as a tumor and metastasis suppressor gene in several types of cancer including breast, pancreatic and prostate cancers^9–12^. Studies have shown that iron and copper chelators exhibited their anti-tumor effects through up-regulating NDRG1 level to regress tumor growth and suppress metastasis^4,13,14^. Moreover, chelators such as those of the dipyridyl thiosemicarbazone (DpT) class also exerted their metastasis-suppressive effects through up-regulating NDRG1^15,16^. In summary, NDRG1 may be a promising therapeutic target for the treatment of cancer.

It was recently discovered that NDRG1 was involved in regulating multiple oncogenic signaling molecules^15,17^. Dixon *et al*. showed that the chelator, Dp44mT exert its anti-proliferative activity by suppressing oncogenic ERK signaling pathway via NDRG1^5^. Moreover, a number of studies showed that the Ras/Raf/MEK/ERK pathway regulates the activity of various oncogenic molecules and is also targeted by NDRG1^18–20^. Recent study also revealed that up-regulation of NDRG1 has an important role in the down-regulation of p-AKT and p-ERK1/2 in prostate cancer cells^5^. And the activity of EGFR as a key receptor tyrosine kinase was shown to be regulated by NDRG1 in human pancreatic cancer cells^20,21^. However, whether NDRG1 may regulate HER2 expression and affect its downstream molecules in HER2-overexpressed cancer cells remains unclear.

Considering the role of NDRG1 in tumor progression, therapeutics that can regulate this molecule remains to be developed. As mentioned above, the thiosemicarbazones displayed the ability on up-regulating NDRG1 level while only a few studies on NDRG1 regulation by dithiocarbamates were conducted. Thus, we prepared a novel dithiocarbamate derivative, dipyridylhydrazone dithiocarbamate (DpdtC) and evaluated its growth inhibitory ability on hepatocellular carcinoma cells in our previous research^3,22^. The preliminary data showed that the DpdtC could inhibit catalase and induce ROS generation^22^. In the present study, we evaluated the anti-tumor effects of DpdtC on HER2-overexpressed cancer cells and investigated involved signaling pathway. Our results revealed that DpdtC potently inhibited the proliferation of SK-OV-3 cells *in vitro* and *in vivo*. Further, data showed that DpdtC exerted the effects through up-regulating NDRG1 level and decreasing HER2 expression. Moreover, we found that silencing endogenous NDRG1 also increased the HER2 level in SK-OV-3 cells, while overexpressing NDRG1 resulted in the down-regulation of HER2 expression in SK-BR-3 cells. More importantly, EGFR/HER2 heterodimer was attenuated and phosphorylation of ERK1/2 was inhibited in response to the EGF treatment. In conclusion, our results suggest that NDRG1 may play an important role in mediating the anti-tumor activity of DpdtC in HER2-overexpressed cancer via selective suppression of the HER2-ERK1/2 pathway. Therefore, DpdtC may be an effective new agent for the treatment of HER2-overexpressed cancers.

## Results

### DpdtC shows the potent inhibitory effects against HER2-overexpressed cancer cells *in vitro*

**Figure 1:**
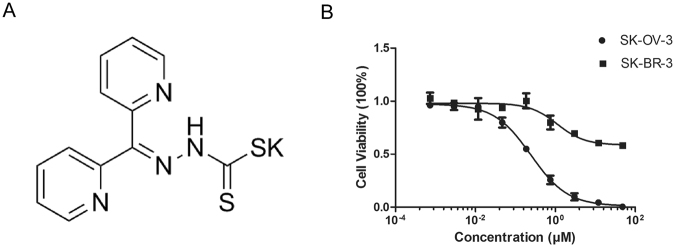
DpdtC inhibited the growth of HER2-overexpressed cancer cells *in vitro*. (**A**) Chemical structure of dipyridylhydrazone dithiocarbamate (DpdtC). (**B**) Inhibitory effect of increasing concentrations of DpdtC on the proliferation of SK-OV-3 or SK-BR-3 cells. The data are shown as the mean ± SD. IC_50_ for SK-OV-3 cells is 0.244 μM (95% CI, 0.211–0.283 μM), whereas IC_50_ of achieving ~50% growth inhibition for SK-BR-3 cells is at ~100 μM. Data were obtained from 3 independent experiments.

We first examined the cytotoxicity of DpdtC against HER2-overexpressed SK-OV-3 and SK-BR-3 cells. Chemical structure of the novel compound was described in Fig. 1A. As depicted in Fig. 1B, DpdtC showed a dose-dependent inhibitory activity in both SK-OV-3 and SK-BR-3 cells. Moreover, SK-OV-3 cells responded more sensitively to DpdtC compared with SK-BR-3 cells.

### DpdtC inhibited the *in vivo* growth of SK-OV-3 cancer xenografts

**Figure 2:**
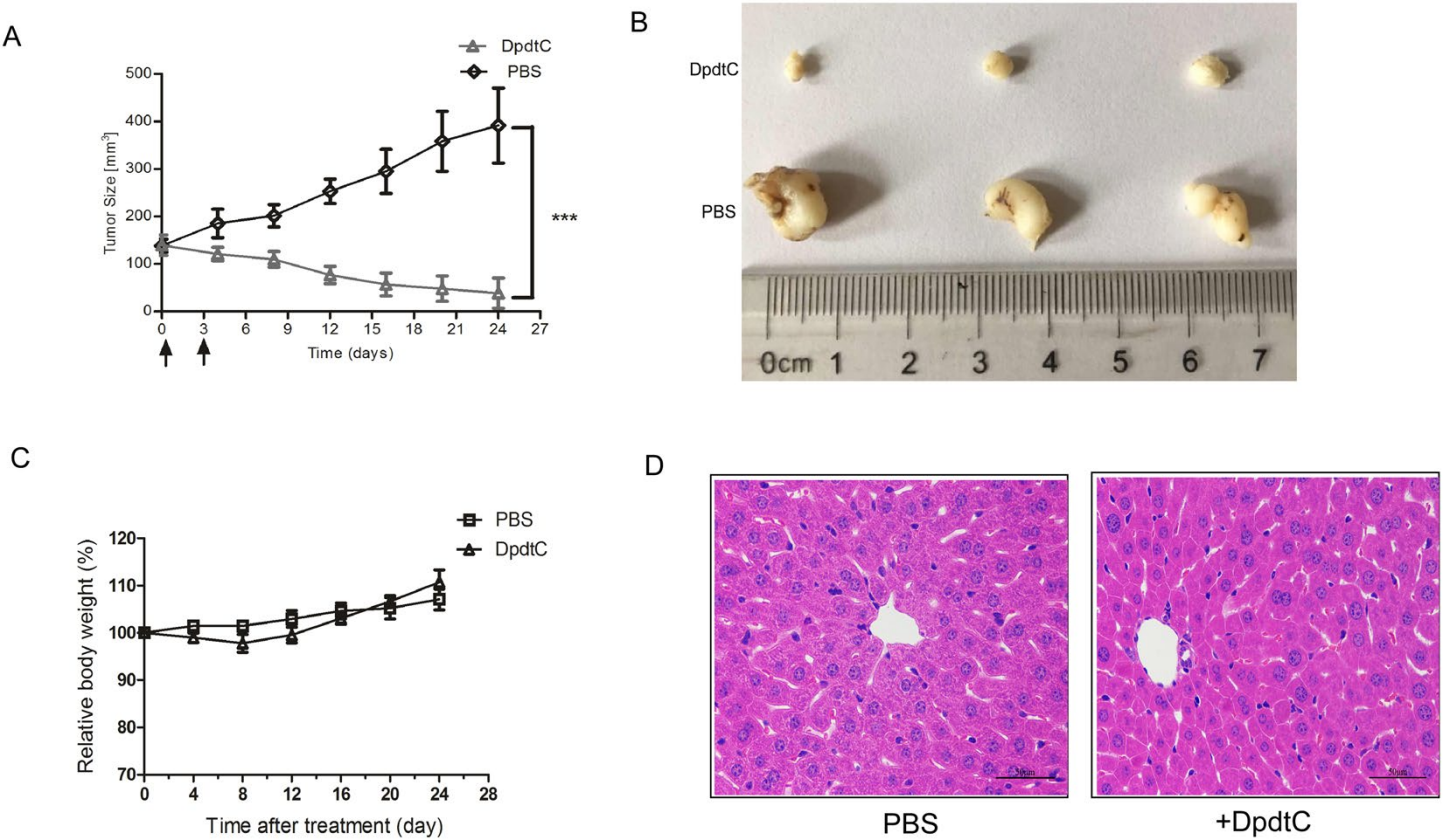
*In vivo* efficacy of DpdtC in the SK-OV-3 xenograft tumor model. (**A**) Mean tumor volumes of mice xenografted with SK-OV-3 cells and treated with DpdtC (5 mg/kg). There were 6 animals per treatment group. DpdtC treatment started as indicated in the graphs (black arrows). Error bars show ± SD. (***P < 0.001). (**B**) On day 24, xenograft tumor from each group were removed and photographed. Representative tumors in each group were shown. (**C**) Effect of DpdtC on nude mice body weight was determined using SK-OV-3 tumor-bearing nude mice. Mice were weighed at regular intervals during the whole period to monitor therapy-related toxicity. (**D**) Histological examination was conducted in nude mice post injection with DpdtC (5 mg/kg) for two times. Images (magnification, ×400) of liver from nude mice (n = 3) injected with PBS (−) or DpdtC (5 mg/kg) for two times were obtained by staining with hematoxylin and eosin. Scale bars, 50 μm.

Next, the therapeutic effects of DpdtC were examined in nude mice bearing established SK-OV-3 xenograft tumors. Results revealed that DpdtC significantly prevented tumor growth compared to control treatment (Fig. 2A,B). To further assess the therapy-related unspecific toxicity on DpdtC treatment, body weight was monitored in nude mice bearing established SK-OV-3 tumor xenografts. As shown in Fig. 2C, treatment with DpdtC was well tolerated and the mean body weight remarkably recovered after marginal weight loss post DpdtC injection. More importantly, hematoxylin & eosin (H&E) staining showed that no marked liver toxicity was observed in DpdtC-treated mice (Fig. 2D). Furthermore, transaminase activity was also examined as hematologic toxicity evaluation index. As shown in supplementary Fig. S1, DpdtC treatment only slightly elevated alanine aminotransferase (ALT) and aspartate aminotransferase (AST) activity in plasma. Thus, our results showed that DpdtC exhibited potent inhibitory effect and good tolerance on SK-OV-3 tumor xenografts.

### DpdtC induced NDRG1 expression and down-regulated the membrane expression of HER2 in SK-OV-3 cells

**Figure 3:**
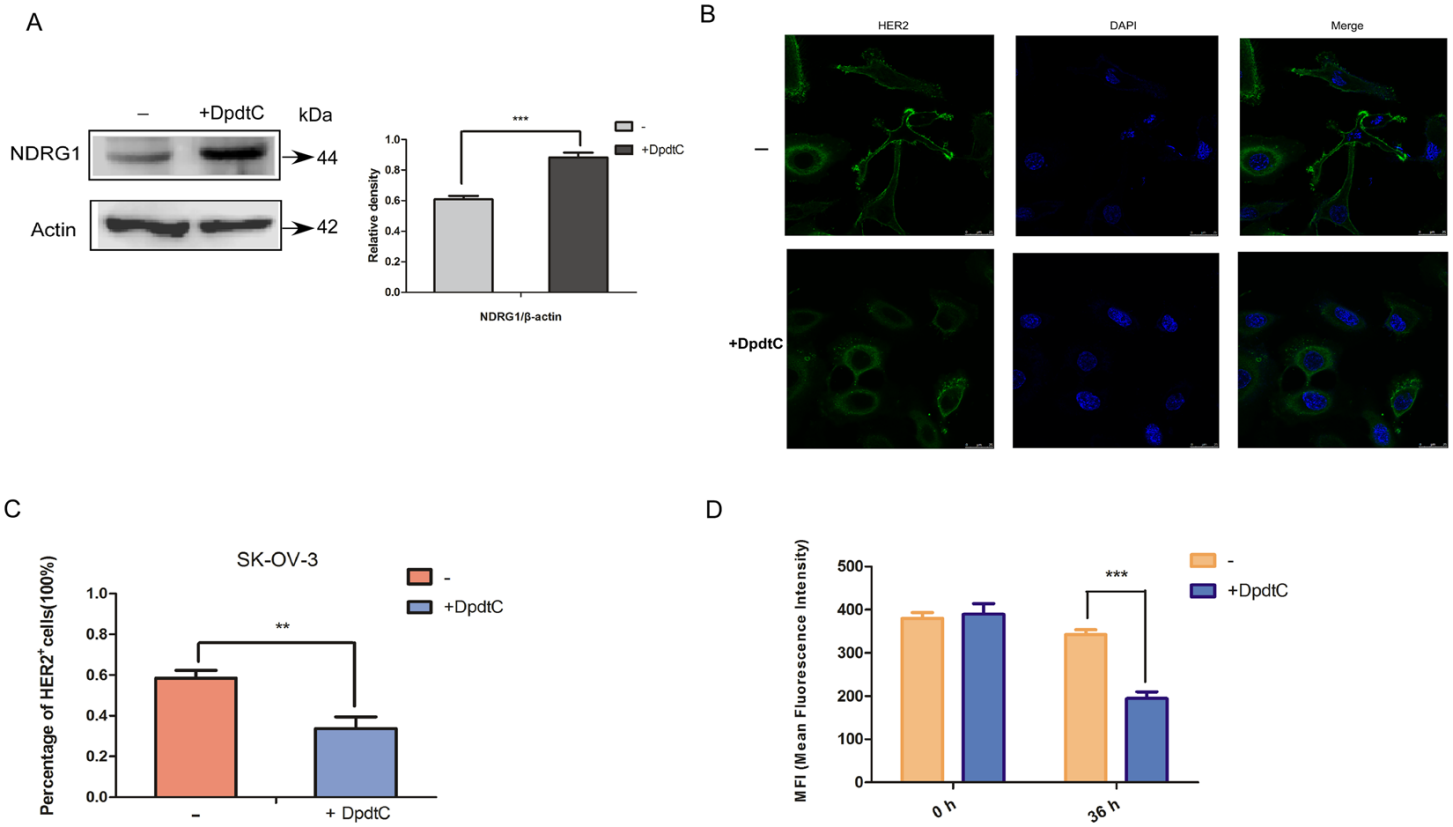
DpdtC up-regulated NDRG1 expression and down-regulated membrane expression of HER2 on SK-OV-3 cells. (**A**) SK-OV-3 cells were incubated for 24 h at 37 °C with control media (−) or media containing the DpdtC (2 μM), and NDRG1 level was examined by western blot. And quantification of western blot signal intensity analysis is expressed relative to the β-actin loading control by using Image J software. ***p < 0.001. (**B**) Representative micrographs of fluorescent immunostaining showed that DpdtC decreased HER2 expression at the cell membrane. Cells were incubated for 36 h at 37 °C with control media (−) or media containing the DpdtC (2 μM), and HER2 localization was examined via immunofluorescence. Original magnification, ×600. Scale bars, 25 μm. (**C**) Percentage of HER2-positive stained cells reduced markedly in SK-OV-3 cells when incubated with DpdtC. Data are expressed as mean ± SD of the integrated fluorescence signals from 3 fields for each specimen. (**D**) Flow cytometry assay quantifying the membrane expression of HER2 on SK-OV-3 cells, which was expressed as MFI (Median Fluorescence Intensity) upon treatment control media (−) or media containing the DpdtC (2 μM) for 36 h at 37 °C. **p < 0.01; ***p < 0.001.

Previous studies have demonstrated that thiosemicarbazones were able to exert anti-tumor effects through up-regulating NDRG1 expression^1,6,23^. In our study, we also found that DpdtC markedly induced NDRG1 expression level (Fig. 3A). More importantly, decreased HER2 expression on the membrane of SK-OV-3 cells was observed by confocal microscope (Fig. 3B). Statistical analysis (Fig. 3C) demonstrated that the percentage of HER2-stained cells upon DpdtC treatment significantly reduced compared to untreatment condition. To further verify the effects of DpdtC on HER2, we also quantified the membrane expression of HER2 on SK-OV-3 cells after treatment with DpdtC by flow cytometry assay. As shown in Fig. 3D, membrane expression of HER2 was down-regulated upon DpdtC treatment. Consequently, these results suggested that DpdtC induced NDRG1 expression, which may suppress HER2 distribution and expression on membrane.

### HER2 expression was regulated by NDRG1 in SK-OV-3 and SK-BR-3 cells

**Figure 4:**
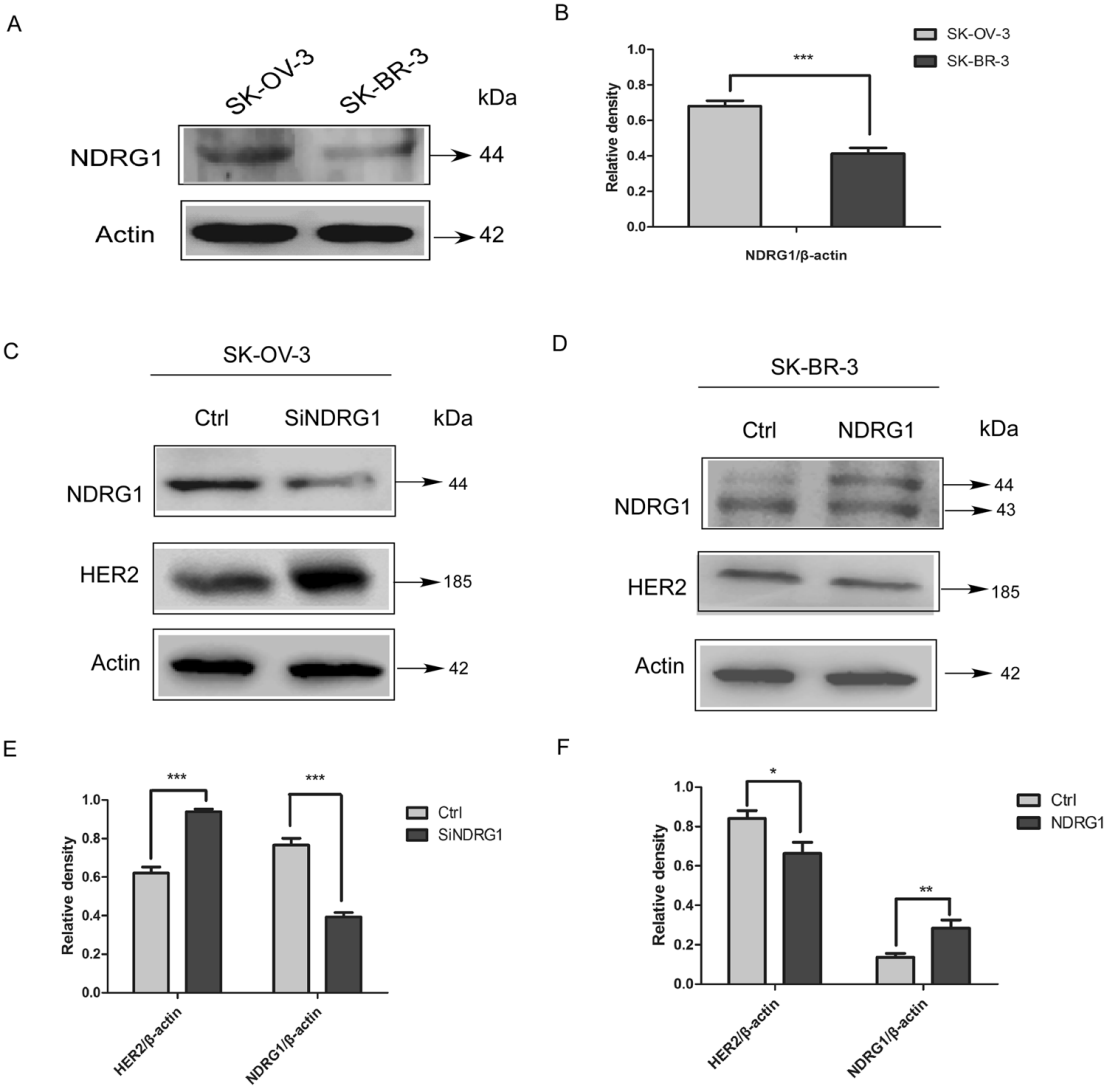
NDRG1 expression decreased HER2 level in both SK-OV-3 and SK-BR-3 cells. (**A**) Western blot indicating the expression of NDRG1 in SK-OV-3 and SK-BR-3 cells. (**B**) Quantification of western blot signal intensity analysis is expressed relative to the β-actin loading control by using Image J software. (**C**) Suppression of NDRG1 by siRNA leaded to increase in the level of HER2 in SK-OV-3 cells. SK-OV-3 cells were transiently transfected with nonspecific control siRNA (Ctrl) or NDRG1 siRNA (siNDRG1) for 72 h at 37 °C. (**D**) NDRG1 expression inhibits HER2 expression in SK-BR-3 cells. The plasmid of pCDNA3.1-NDRG1 or the vector control (Ctrl) was constructed and transfected into SK-BR-3 cells. (**E**) Quantification of western blot signal intensity analysis in SK-OV-3 cells is expressed relative to the β-actin loading control by using Image J software. (**F**) Quantification of western blot signal intensity analysis in SK-BR-3 cells is expressed relative to the β-actin loading control by using Image J software. Data show the mean ± SD (3 independent experiments); *p < 0.05; **p < 0.01; ***p < 0.001.

To determine how NDRG1 could be involved in the effects of DpdtC on HER2 level, we silenced NDRG1 expression in SK-OV-3 cells using the screened siRNA (Fig. S2). SK-OV-3 cell line was chosen for the study due to the relatively higher expression of NDRG1 compared to SK-BR-3 cells (Fig. 4A,B). This may partly explained the difference in how SK-OV-3 and SK-BR-3 cells respond to DpdtC (Figs 4A and S3). As shown in Figs 4C,E and S4, silencing NDRG1 resulted in significantly increased HER2 level (P < 0.001), while no significant change in the level of β-actin was observed. In contrast, overexpression of NDRG1 in SK-BR-3 cells resulted in significantly reduced HER2 expression (P < 0.05) (Fig. 4D,F). In summary, these results revealed that NDRG1 was able to antagonize HER2 expression in HER2-amplified SK-OV-3 and SK-BR-3 cells.

**Figure 5:**
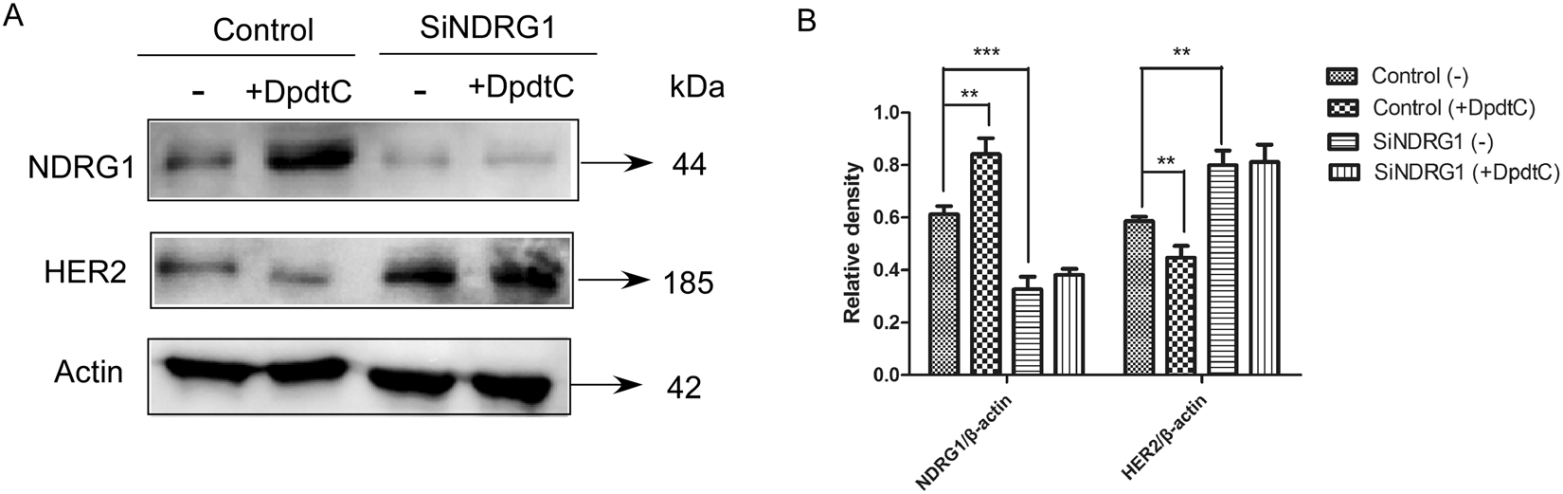
Effect of DpdtC on suppressing HER2 expression is dependent on NDRG1 in SK-OV-3 cells. (**A**) SK-OV-3 cells were transfected with control siRNA or siNDRG1 for 72 h at 37 °C, followed by treatment with DpdtC (2 μM) for 30 h at 37 °C. Then levels of NDRG1 and HER2 were tested by western blot. (**B**) Quantification of western blot signal intensity analysis is expressed relative to the β-actin loading control by using Image J software. Data show the mean ± SD (3 independent experiments); **p < 0.01; ***p < 0.001.

To further investigate that HER2 was affected by DpdtC via NDRG1, we assessed the effect of DpdtC on HER2 expression when NDRG1 was silenced in SK-OV-3 cells. Results showed that DpdtC has no marked effects on inducing NDRG1 expression and inhibiting the expression of HER2 when NDRG1 was silenced in SK-OV-3 cells (Fig. 5A,B). Hence, our results suggested that the effects of DpdtC on down-regulating HER2 level may be dependent on NDRG1.

### NDRG1 down-regulates HER2 and EGFR level and inhibits the formation of HER2/EGFR heterodimer

**Figure 6:**
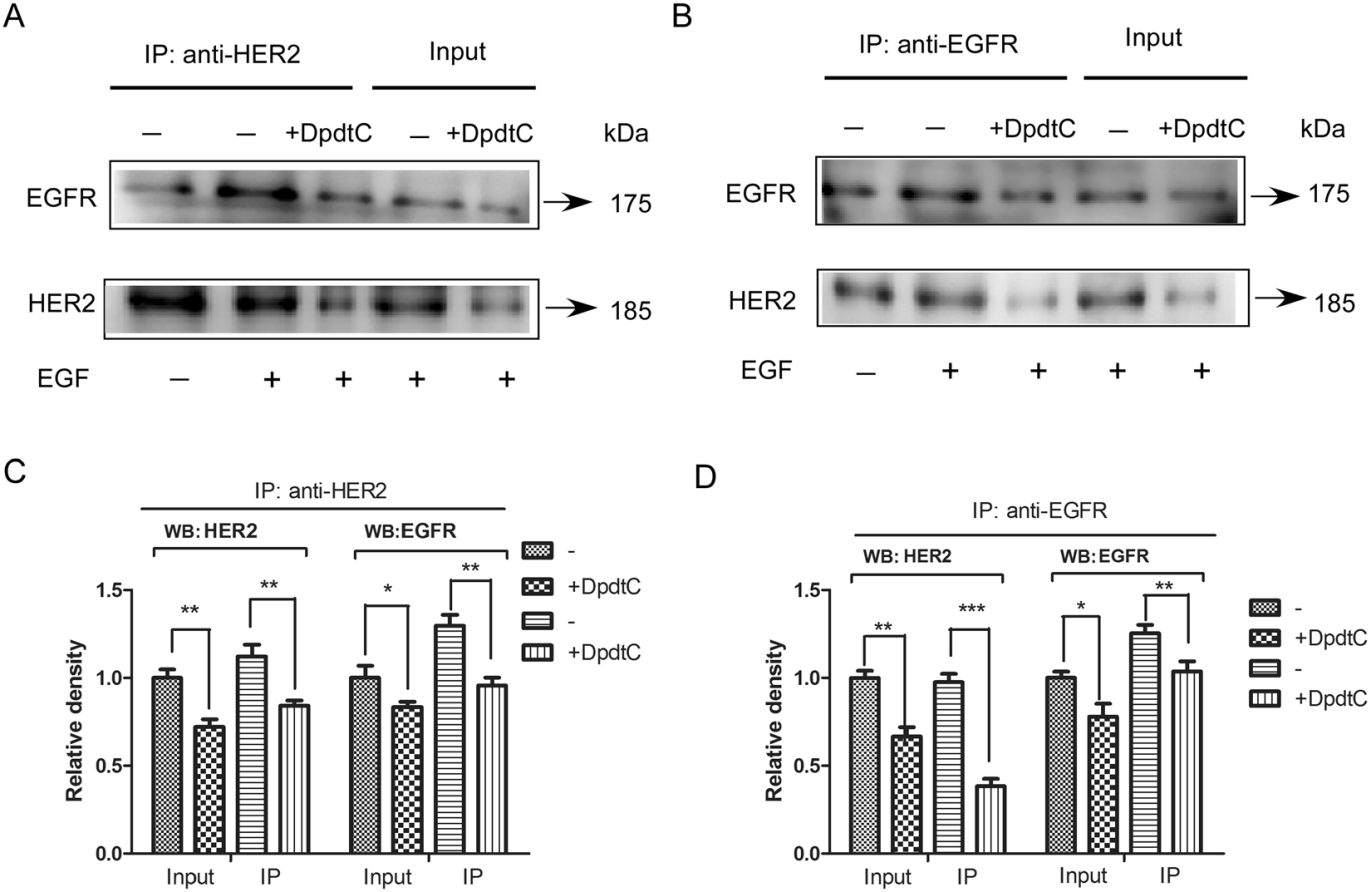
HER2/EGFR heterodimer formation was inhibited in SK-OV-3 cells when treated with DpdtC. (**A** and **B**) SK-OV-3 cells were incubated with control medium (−) or medium with DpdtC (2 μM) for 36 h at 37 °C in the absence or presence of EGF, and tested via co-immunoprecipitation according to “Materials and Methods”. (**C** and **D**) Quantification of western blot signal intensity analysis is expressed relative to untreated control cells by using Image J software. Data show the mean ± SD (3 independent experiments); *p < 0.05; **p < 0.01; ***p < 0.001.

HER2 forms heterodimer with EGFR and triggers activation of key downstream signaling molecules involved in HER2-overexpressed cancer cell proliferation and survival^24,25^. Considering these, we investigated whether formation of HER2/EGFR heterodimer was affected by NDRG1 in SK-OV-3 cells upon treatment with DpdtC in the presence of EGF ligand. SK-OV-3 lysates were co-immunoprecipitated with anti-HER2 antibody, and then EGFR and HER2 levels were tested by western blot. Results showed that EGFR/HER2 heterodimer was significantly reduced in SK-OV-3 cells treated with DpdtC compared to untreated group (Fig. 6A,C). In agreement with these results, decreased EGFR/HER2 heterodimer was also observed in DpdtC-treated cells when co-immunoprecipitated with anti-EGFR antibody (Fig. 6B,D). Consequently, our study demonstrated that elevated NDRG1 level induced by DpdtC resulted in reduced HER2 and EGFR level, which may further lead to the decreased HER2/EGFR heterodimer formation.

### NDRG1 inhibits the phosphorylation of ERK1/2 in response to EGF

**Figure 7:**
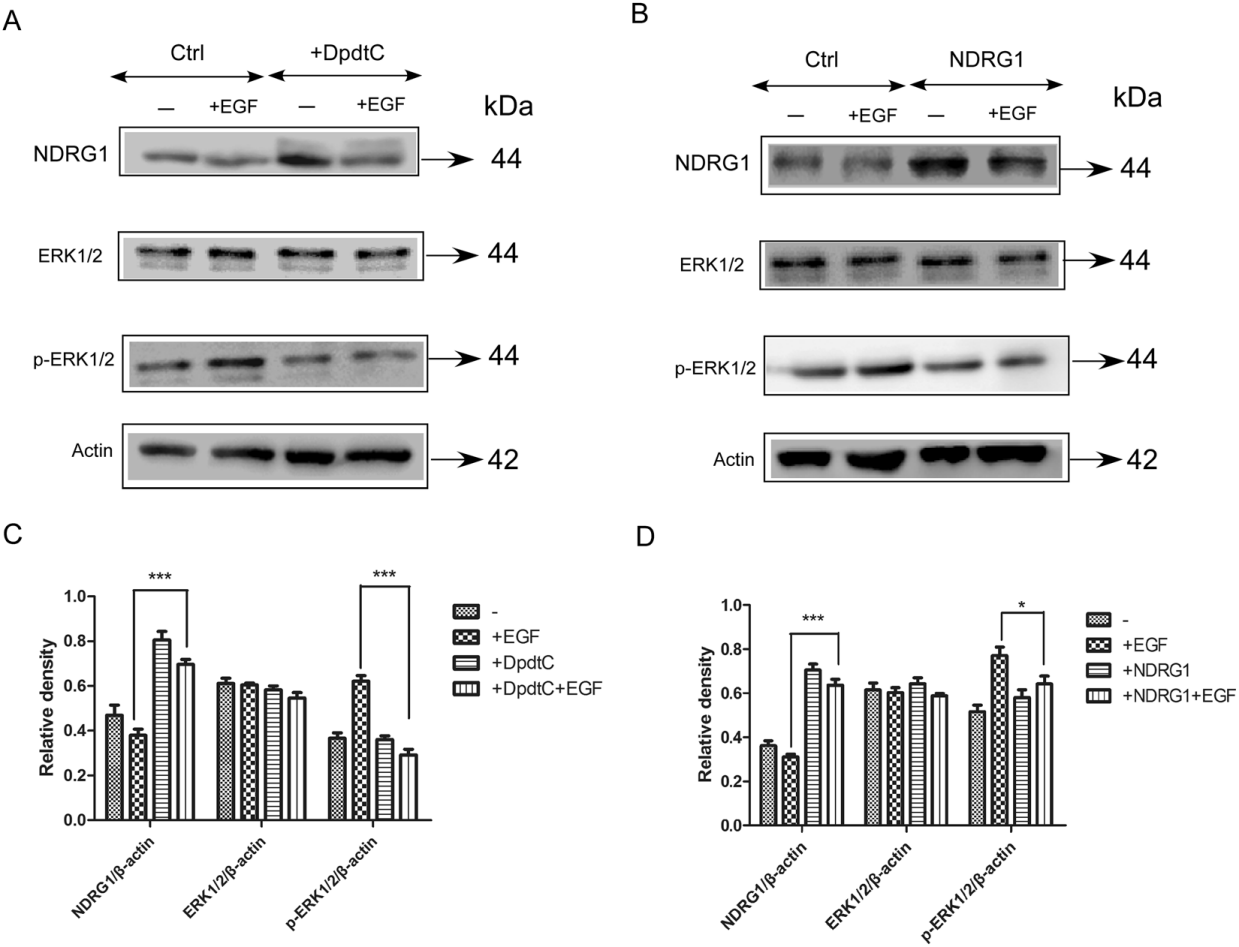
Up-regulation of NDRG1 inhibited HER2-ERK1/2 downstream signaling pathway in SK-OV-3 cells. (**A**) SK-OV-3 cells were incubated with control medium(−) or medium containing DpdtC for 36 h at 37 °C followed by treatment with EGF (10 ng/ml) in the last 20 min of 36 h incubation. (**B**) Vector control (Ctrl) or NDRG1-overexpressing (NDRG1) cells were incubated with control medium(−) or medium containing EGF (10 ng/ml; 20 min/37 °C) and levels of NDRG1, ERK 1/2,pERK 1/2 were tested by western blot. (**C** and **D**) Quantification of western blot signal intensity analysis is expressed relative to β-actin by using Image J software. Data show the mean ± SD (3 independent experiments); *p < 0.05; ***p < 0.001.

As we know, phosphorylation of ERK1/2 was important for activation of ERK1/2 MAPK signaling pathway^26,27^. Then the effect of NDRG1 on the activation of the key downstream molecule ERK1/2 was investigated. First, we observed that increased NDRG1 induced by DpdtC treatment resulted in significant regression of p-ERK1/2 level (Fig. 7A,C). Furthermore, NDRG1 overexpression in SK-OV-3 cells also caused a significant decrease on p-ERK1/2 level, while no significant alteration was found on total ERK1/2 level (Fig. 7B,D). To conclude, our results showed that both NDRG1 overexpression and treatment with DpdtC caused significant inhibitory effect on HER2/EGFR heterodimer formation and ERK1/2 activation, which may be a novel mechanism involved in the potent anti-tumor activity of DpdtC.

## Discussion

Metal chelators including thiosemicarbazones and dithiocarbamates were found to have effective and selective anti-tumor activity against various different cancers^3,28–30^. Studies revealed that thiosemicarbazones such as Dp44mT, DpC and DFO exhibited their anti-proliferative activity through blocking multiple signaling pathways involved in tumorigenicity and metastasis^1,17,31^. Dithiocarbamates are a group of sulfur-containing compounds with a strong chelating ability toward metal ions^8,22^. Previous studies revealed that dithiocarbamate derivatives may act as nuclear factor kappa B (NF-κB) inhibitors^32^, proteasome inhibitors^33^, DNA intercalators^34^, and inactivators of various metal-containing enzymes^35^; however, the detailed mechanism of action remains largely unclear.

Chemical properties of the new dithiocarbamate compound, DpdtC have been characterized in our previous study^3^. In the study, we for the first time reported the anti-tumor effects of DpdtC in HER2-overexpressed cancer cells. Previous studies have revealed that iron chelators could induce iron depletion in cancer cells and the decrease in cellular iron may lead to the robust up-regulation of NDRG1^31,36^. Our study demonstrated that DpdtC exhibited potent anti-tumor effects both *in vitro* and *in vivo* through up-regulating NDRG1 level, which was consistent with the studies mentioned above.

NDRG1 has been found to be involved in several oncogenic signaling pathways. Dixon *et al*. showed that treatment with the iron chelator, Dp44mT inhibited AKT, TGF-β and ERK signaling pathway through up-regulating NDRG1 level in prostate cells^5^. Recently, Kovacevic *et al*. also revealed that Dp44mT induced NDRG1 level and inhibited ErbB family receptors related oncogenic signaling pathway in human pancreatic cancer cells^21^. It has been suggested that NDRG1 could play a role in promoting receptor degradation, which partly explained the molecular mechanism by which NDRG1 inhibited ErbB family receptor^21^. To our knowledge, the mitogen-inducible factor 6 (MIG6), which is also known as ErbB receptor feedback inhibitor, could increase EGFR internalization and trafficking to the lysosome. MIG6 was upregulated when cells were treated with iron or copper chelators^37–39^. In our study, we also observed HER2 was repressed by DpdtC stimulated up-regulation of NDRG1 in HER2-overexpressed SK-OV-3 cells, and the mechanism will be further explored in the following studies.

Despite as the potent metastasis suppressor, how NDRG1 participates in the mechanism of dithiocarbamate-mediated tumor growth inhibition remains unclear. In our research, we found that NDRG1 plays an important role in mediating the anti-tumor effects of DpdtC in HER2-overexpressed cancer cells through inhibiting the formation of HER2/EGFR heterodimer. The HER2/EGFR heterodimer is crucial for HER2-mediated signaling pathways in tumors containing amplifications of HER2^25,40^. And therapeutic agents that disrupted HER2/EGFR heterodimer will result in PI3K/AKT or MEK/ERK1/2 pathway downstream signaling interference^26,41^. Kovacevic *et al*. have revealed that NDRG1 inhibited the formation of heterodimer among EGFR, HER2 and HER3 to regress downstream oncogenic signaling^21^. In agreement with these results, our data revealed that up-regulation of NDRG1 induced by DpdtC treatment also decreased HER2 expression, reduced the formation of HER2/EGFR heterodimer and then inhibited the activation of HER2-ERK1/2 pathway in HER2-overexpressed SK-OV-3 cells. As we know, HER2 was an important membrane receptor that various agents including antibodies and small molecules were designed to target^42,43^. Hence, these results above suggested that combination of HER2-targeted agents with NDRG1 inducer, DpdtC may achieve greater anti-tumor effects via more effectively inhibiting the phosphorylation of ERK1/2 and downstream signaling pathway.

Collectively, the findings in our study demonstrated that the promising novel anti-tumor agent, DpdtC that can up-regulate NDRG1 and target HER2-ERK1/2 pathway, has the potential to provide a new therapeutic strategy in HER2-overexpressing cancer treatment.

## Materials and Methods

### Cell lines

The human ovarian cancer cell line SK-OV-3 and breast cancer cell line SK-BR-3 were purchased from the American Type Culture Collection (ATCC).

### Animals

All experimental protocols were approved by the Animal Experimentation Ethics Committee of Xinxiang Medical University and all efforts were made to minimize animal suffering and reduce the number of animals used. All experiments were performed in accordance with the guideline of the Animal Care and Use Committee of Xinxiang Medical University. Five-week-old female BALB/c nude mice were obtained from the Beijing Vital River Laboratory Animal Technology Co., Ltd. (Beijing, China).

### Immunoprecipitation

To detect the HER2/EGFR heterodimer, immunoprecipitation assay was performed as mentioned below. Briefly, cells were washed with ice-cold PBS and lysed using the lysis buffer (Beijing Dingguo Biotechnology Co. Ltd, Beijing) containing protease inhibitors. Protein (600 μg) was incubated with either anti-HER2 antibody (5 μg, Sc-7301; Santa Cruz) or anti-EGFR antibody (5 μg, Sc-03-G; Santa Cruz) overnight at 4 °C. This mixture was added to 20 μL of Protein A (Zhangjiang Biotech Inc, Shanghai) and incubated for 6 h at 4 °C. The beads were then washed three times with ice-cold PBS, and then samples were separated on a 10% gel. HER2 and EGFR were detected by western blot assay.

### ***In vitro*** cytotoxity assays

Cells were incubated with increasing concentrations of DpdtC. Two days later, cell proliferation was determined using Cell Counting Kit 8 (CCK-8) kit (Dojindo, Japan). The percentage of surviving cells was calculated using the following formula: [(A450 of experiment - A450 of background)/(A450 of untreated control - A450 of background)] × 100.

### ***In vivo*** therapy study and toxicities evaluation

SK-OV-3 cells (5 × 10^6^ per mouse) were inoculated subcutaneously into the right flank of female BALB/c nude mice. When tumor volumes reached an average of about 150 mm^3^, the mice were randomly divided into 2 groups of 6 mice each. Mice were intraperitoneally injected with PBS or DpdtC (5 mg/kg) for two times as indicated. Tumors were measured with digital calipers, and tumor volumes were calculated by the formula: volume = length × (width)^2^/2. Toxicities evaluation was determined in nude mice post intraperitoneal injection of DpdtC for two times at 5 mg/kg body weight. Then toxicities were assessed by body weight change, histological examination, hematologic index evaluation.

### Immunoblotting

Western blot was performed using established procedures^40^. Cells were lysed in lysis buffer (Beijing Dingguo Biotechnology Co. Ltd), incubated on ice for 30 min and centrifuged for 20 min to remove cell debris. Total cell lysates were subjected to SDS–polyacrylamide and immunoblotted with antibodies against EGFR (Sc-03-G; Santa Cruz), p44/42 ERK (9102; Cell Signaling), phospho-p44/42 ERK-Thr202/Tyr204 (9106; Cell Signaling), NDRG1 (5196; Cell Signaling), HER2 (Sc-7301; Santa-Cruz), β-actin (ab1801; Abcam).

### Immunofluorescence

The assay was performed as described previously^44^. Cells were seeded on the chamber slide and fixed with ice cold 4% paraformaldehyde in PBS for 20 min and blocked with 1% BSA for one hour. Cells were stained for indicated primary antibody and secondary antibody, and then visualized using a Leica® confocal microscopy system.

### Flow Cytometric analysis

SK-OV-3 cells treated with DpdtC or control were incubated on ice with anti-HER2 antibody in fluorescence activated cell sorting (FACS) buffer (PBS buffer containing 1% fetal bovine serum) for 1 hour. Cells were washed with FACS buffer and incubated with FITC labeled IgG (H + L) secondary antibody on ice for 30 min. Then all samples were washed using FACS buffer and followed by FACS analysis using a BD FACSCalibur system (BD Biosciences).

### Statistical analysis

Statistical analysis was performed by Student’s unpaired t test to identify significant differences unless otherwise indicated. Differences were considered significant at P < 0.05.

### Data availability

The authors declare that all data supporting the findings of this study are available within the article and its supplementary data files, or are available from the corresponding author upon request.

## Electronic supplementary material

Supplementary Data
