# Supplementary material for: The novel dithiocarbamate, DpdtC suppresses HER2-overexpressed cancer cells by up-regulating NDRG1 via inactivation of HER2-ERK 1/2 signaling
Source: Sci Rep. 2018 Feb 21;8:3398. doi: 10.1038/s41598-018-21768-1 (PMC5821706; doi:10.1038/s41598-018-21768-1)
Supplement: Supplementary file 1 — Supplementary Data [file 41598_2018_21768_MOESM1_ESM.pdf]

The novel dithiocarbamate, DpdtC suppresses HER2-overexpressed cancer cells by up-regulating NDRG1 via inactivation of HER2-ERK 1/2 signaling

Yun Yang[1,*], Youxun Liu[1], Rui Guo[2], Yun Fu[1], Ziheng Zhang[1], Pengfei Zhang[1], Pingxin Zhou[1], Tingting Wang[1], Tengfei Huang[1], Xiaotong Li[3] and Changzheng Li[1,*]

[1] School of Basic Medical Sciences, Xinxiang Medical University, Xinxiang, China.

[2] College of Biomedical Engineering, Xinxiang Medical University, Xinxiang, China.

[3] School of Life Sciences, Xiamen University, Xiamen, China.

*Corresponding Author. E-mail address: Yun Yang, E-mail: yangyun@xxmu.edu.cn; Changzheng Li, E-mail: changzhengli@xxmu.edu.cn;

Supplementary Data

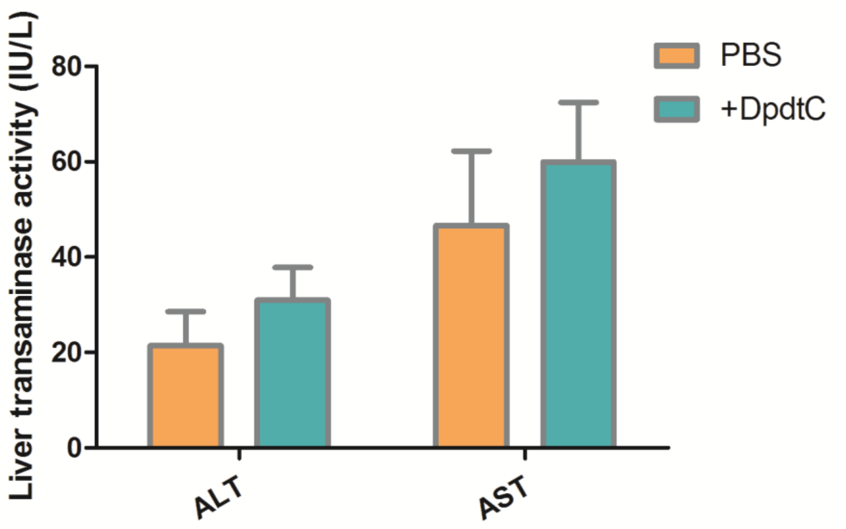

Figure S1. Liver alanine amino transaminase (ALT) and aspartate aminotransferase (AST) activity were determined in the plasma from nude mice (n=3) treated with DpdtC or PBS. No significant difference on ALT or AST level was found between DpdtC-treated group and PBS-treated group. Error bars show ± SD.

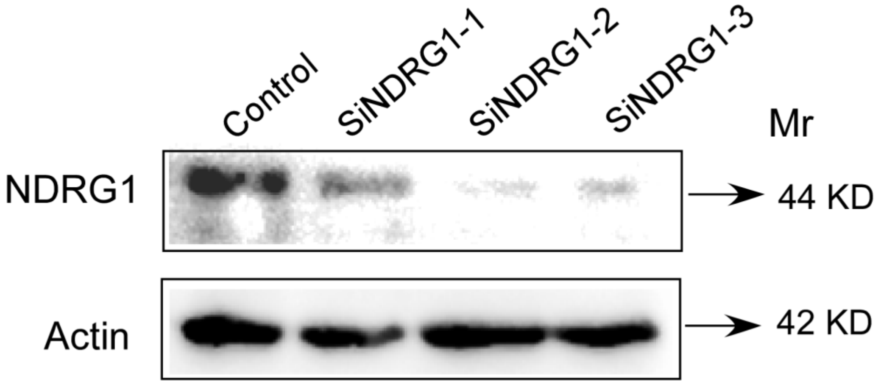

Figure S2. Three SiRNA expression constructs targeting different region of NDRG1 sequence were tested by immunoblotting. Then, SiNDRG1-2 was chosen for the following assays due to its pronounced interference effect.

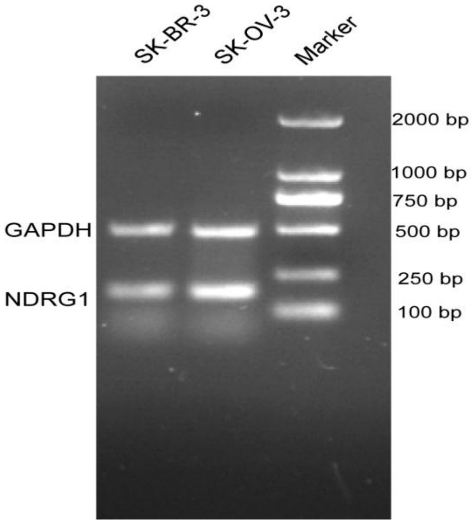

Figure S3. The RNA level of SK-BR-3 and SK-OV-3 were evaluated by semi-quantitative RT-PCR analysis.

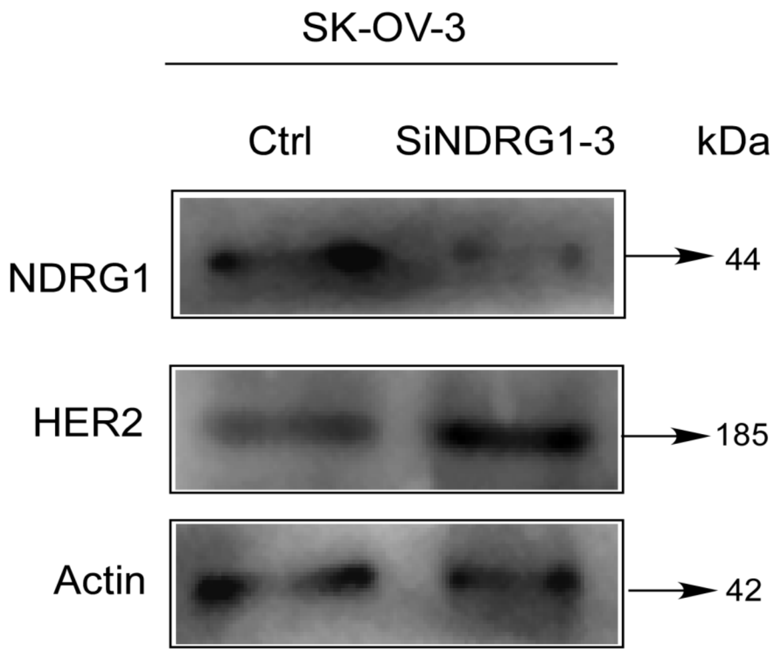

Figure S4. SiNDRG1-3, which was another siRNA sequence against NDRG1, was used to verify the inhibitory effects of NDRG1 on HER2 level in SK-OV-3 cells.
